# Identification and evaluation of the role of the manganese efflux protein in *Deinococcus radiodurans*

**DOI:** 10.1186/1471-2180-10-319

**Published:** 2010-12-14

**Authors:** Hongxing Sun, Guangzhi Xu, Hongdan Zhan, Huan Chen, Zongtao Sun, Bing Tian, Yuejin Hua

**Affiliations:** 1 Key Laboratory for Nuclear-Agricultural Sciences of Chinese Ministry of Agriculture and Zhejiang Province, Institute of Nuclear-Agricultural Sciences, Zhejiang University, 310029, Hangzhou, PR China; 2 Zhejiang Institute of Microbiology, Zhejiang Province, Hangzhou, 310012, PR China

## Abstract

**Background:**

*Deinococcus radiodurans *accumulates high levels of manganese ions, and this is believed to be correlated with the radiation resistance ability of this microorganism. However, the maintenance of manganese ion homeostasis in *D. radiodurans *remains to be investigated.

**Results:**

In this study, we identified the manganese efflux protein (MntE) in *D. radiodurans*. The null mutant of *mntE *was more sensitive than the wild-type strain to manganese ions, and the growth of the *mntE *mutant was delayed in manganese-supplemented media. Furthermore, there was a substantial increase in the *in vivo *concentration of manganese ions. Consistent with these characteristics, the *mntE *mutant was more resistant to H_2_O_2_, ultraviolet rays, and γ-radiation. The intracellular protein oxidation (carbonylation) level of the mutant strain was remarkably lower than that of the wild-type strain.

**Conclusions:**

Our results indicated that *dr1236 *is indeed a *mntE *homologue and is indispensable for maintaining manganese homeostasis in *D. radiodurans*. The data also provide additional evidence for the involvement of intracellular manganese ions in the radiation resistance of *D. radiodurans*.

## Background

*Deinococcus radiodurans *is an extreme bacterium known for its resistance to ionizing radiation (IR), ultraviolet (UV) radiation, oxidative stress, and desiccation [1,2]. It has been reported that *D. radiodurans *can recover from exposure to γ-radiation at 15 kGy, a dose lethal to most life forms. IR can directly damage biomacromolecules and can also produce reactive oxygen species (ROS) that can indirectly attack both proteins and DNA [3,4]. Therefore, cellular defense against ROS-induced protein and DNA damage is proposed to be important to the radiation resistance of *D. radiodurans *[5].

Manganese plays an important role in the antioxidant systems of bacteria and can relieve the phenotypic deficit of *sod*-null *Escherichia coli *[6]. Interestingly, Daly and coworkers found that the Mn/Fe ratio of most IR-resistant bacteria is higher than that of IR-sensitive bacteria. The group also found that *D. radiodurans *grown in manganese-deficient medium was relatively more sensitive to IR than the bacteria grown in manganese-containing medium, suggesting that the accumulation of intracellular manganese ions can protect proteins from ROS-induced damage and can help in the survival of *D. radiodurans *in extreme environments [5,7,8].

Although manganese can improve cellular ROS resistance, excess manganese is toxic to cells. Thus, maintenance of the intracellular Mn concentration homoeostasis is a challenge. In bacteria, two main classes of manganese transporters have been identified--Nramp H+-Mn^2+ ^transporters and the ATP-binding cassette (ABC) Mn^2+ ^permeases [9]. Recently, a manganese efflux system was identified in *Streptococcus pneumoniae*, and this was found to play important roles in host pathogenesis and H_2_O_2 _resistance [10]. Many genes involved in the maintenance of manganese ion homeostasis have been reported in *D. radiodurans*, such as *dr1709*, *dr2523 *[11], *dr2539 *[12], and *dr0615 *[13]. Therefore, it would be very interesting to determine whether *D. radiodurans *possesses a similar manganese efflux system.

In this study, we identified a manganese efflux gene (*dr1236*) in *D. radiodurans *and demonstrated that it plays an important role in maintaining the homeostasis of intracellular Mn. The null mutant *mntE*^- ^was highly sensitive to manganese ions. When the intracellular level of manganese ions was increased by mutating *dr1236*, the mutant showed clearly enhanced resistance to oxidative stress. Our results also demonstrated that increased intracellular Mn levels could substantially suppress protein oxidation (carbonylation) in *D. radiodurans *exposed to H_2_O_2_, indicating that manganese transport and regulation may be involved in the cellular resistance of *D. radiodurans *to oxidative stress.

## Results and discussion

### D. radiodurans encodes a putative manganese efflux protein

**Figure 1:**
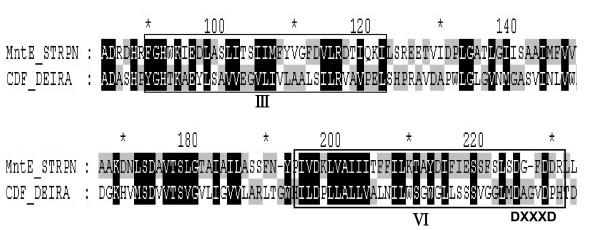
**Sequence alignment of the two manganese efflux proteins**. DEIRA, *Deinococcus radiodurans *R1; STRPN, *Streptococcus pneumoniae*. The metal transfer regions III and VI are boxed. Identical amino acids and similar amino acids are denoted by black and gray backgrounds, respectively.

By searching the *D. radiodurans *genome http://www.ncbi.nlm.nih.gov/, we identified a manganese efflux protein homologue that was annotated as the conserved hypothetical protein DR1236 based on its extensive sequence similarity (25% identity, 49% similarity) to the manganese efflux protein (sp1552) of *Streptococcus pneumoniae *[10]. Similar to most cation diffusion facilitator (CDF) proteins, DR1236 has six putative transmembrane domains (TMDs) http://www.ch.embnet.org/software/TMPRED_form.html. The most conserved region of the CDF protein is the TMD region, which is probably involved in metal transfer [14]. Sequence alignment was performed with the CLUSTAL W program available on the EMBL web page http://www.ebi.ac.uk. The alignment Sp1552 and DR1236 revealed the presence of highly conserved sequences in metal transfer regions III and VI (Figure 1). Moreover, the DXXXD motif, which is conserved in the manganese efflux protein, was also present in DR1236 (224 DAGVD 230).

### mntE is essential for the manganese resistance of D. radiodurans

**Figure 2:**
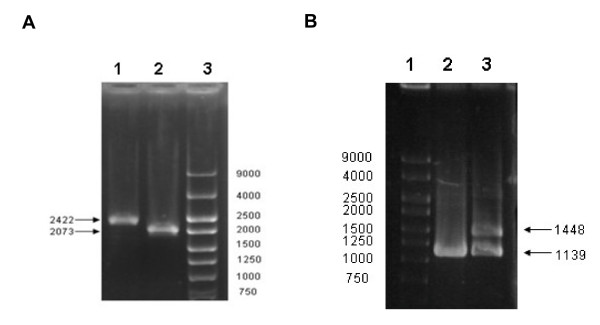
***mntE*^- ^mutant construction and verification by PCR**. **(A) **Ethidium-bromide-stained agarose gel illustrating that the mutant carries a homozygous deletion of *dr1236::aadA*. Lane 1, *mntE*^- ^mutant; lane 2, R1; lane 3, DNA marker. Primers M1/M4 were used for PCR. **(B) **Verification of wild-type revertant *mntE *by PCR. Lane 1, DNA marker; lane 2, R1; lane 3, revertant *mntE*. Primers M5/M6 were used for PCR.

**Figure 3:**
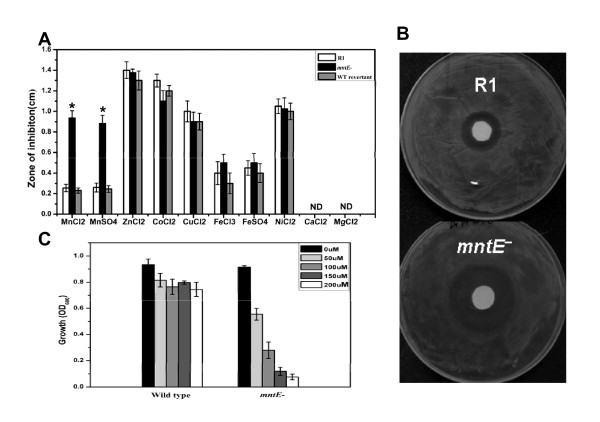
**Manganese sensitivity assay for wild-type R1 and the *mntE*^- ^mutant**. **(A) **Wild-type R1 (white bars), *mntE*^- ^(black bars), and WT revertant (gray bars) were cultured on TGY plates overlaid with filter discs saturated with 1 M solutions of various cations. The zone of inhibition was measured from the edge of the disc after three days. *P < 0.01. ND, not determined. **(B) **The inhibition zone of R1 and *mntE^-^*. Cells were cultured on TGY plates overlaid with filter discs saturated with 1 M manganese chloride. **(C) **Wild-type R1 and *mntE*^- ^were inoculated in TGY supplemented with different concentrations of manganese chloride. The OD_600 _values were determined after 12 h. Data represent the means ± standard deviations of three independent experiments.

To confirm the specific substrate and roles of DR1236 in *D. radiodurans*, the null mutant of *dr1236 *(*mntE^-^*) and wild-type revertant *mntE *strains were constructed (Figure 2). Metals including manganese are essential yet potentially toxic to bacteria [15]. Supplementation with certain metal ions can inhibit the growth of an exporter system mutant [16,17]; therefore, this phenotype is used to verify certain mutants. In this study, wild-type R1 and *dr1236 *(*mntE^-^*) were grown on TGY plates overlaid with discs saturated with 10 μL of different metal ion solutions (1 M) containing manganese, magnesium, cobalt, calcium, copper, zinc, nickel, or iron ions. As shown in Figure 3A/B, the growth of the *mntE*^- ^mutant was strongly inhibited by the manganese ions, but the mutant grew normally in the presence of other cations. Moreover, the wild-type revertant showed a growth phenotype similar to that of R1, indicating that growth inhibition of the *mntE*^- ^mutant was due to the interruption of *dr1236*.

To further investigate the influence of manganese ions on the *mntE*^- ^mutant, different concentrations of manganese ions were added to TGY medium, and the growth of the *mntE^- ^mutant *was measured (Figure 3C). The results showed that in comparison with R1, the growth of the *mntE*^- ^mutant was clearly delayed in the presence of low concentrations of manganese ions. When the manganese concentration increased, the growth defect phenotype became more pronounced. This phenotype is similar to that observed in Rosch's study in which the growth of *S. pneumoniae *having a disrupted calcium efflux system was more severely inhibited at higher calcium concentrations [18].

### The mntE^- ^mutant shows high intracellular manganese concentrations

**Figure 4:**
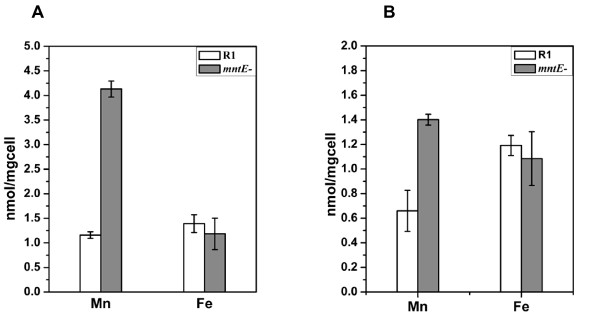
**Analysis of the intracellular ion content of wild-type R1 and *mntE*^- ^cultured in medium supplemented with or without cations**. **(A) **R1 (white bars) and *mntE*^- ^(grey bars) were cultured in TGY medium supplemented with 50 μM manganese, 10 μM ferric chloride, 100 mM magnesium, or 100 mM calcium chloride to determine the effects of these specific cations. **(B) **R1 (white bars) and *mntE*^- ^(grey bars) were cultured in TGY medium without added cations. Cells (OD_600 _= 0.8) were harvested, and the extracellular cations were removed by washing in EDTA. The cation concentration was determined by ICP-MS. The data represent the means ± standard deviations of three independent experiments.

To confirm that the *mntE*^- ^mutant had lost its ability to export manganese ions, the intracellular manganese ion levels of wild-type R1 and the *mntE*^- ^mutant were measured by inductively coupled plasma-mass spectrometry (ICP-MS). As expected, when grown on TGY medium supplemented with manganese ions, the manganese ion level in the *mntE*^- ^mutant was almost four-fold higher than that in wild-type R1. However, there was no significant difference in the intracellular Fe ion concentrations of R1 and the mutant (Figure 4A). Similar results were obtained when the *mntE*^- ^mutant and wild-type R1 were grown on TGY medium (Figure 4B). This result indicates that Dr1236 is a manganese ion exporter.

### The mntE^- ^mutant shows higher resistance to γ-radiation, UV, and oxidative

**Figure 5:**
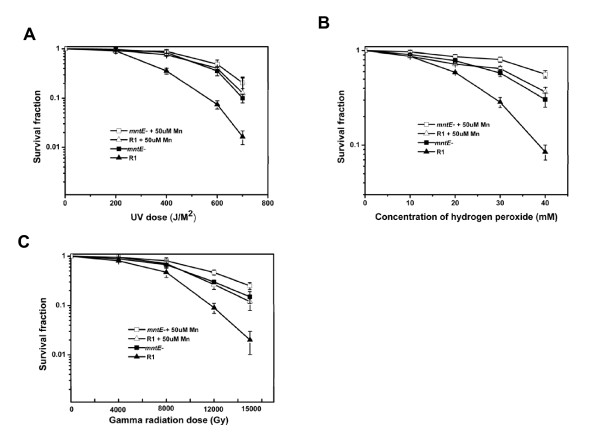
**Survival curves for R1 (triangles) and *mntE*^- ^(squares) following exposure to UV (A), H_2_O_2 _(B), and γ-radiation (C)**. R1 and *mntE*^- ^were cultured in TGY broth with or without 50 μM manganese. The values represent the means ± standard deviations of four independent experiments.

Recently, there has been a debate on whether the high intracellular Mn/Fe ratio of *D. radiodurans *contributes to the extreme oxidative resistance of this microorganism. Daly *et al *proposed that the high Mn/Fe ratio can effectively suppress protein carbonylation and increase radiation resistance [7,8]. In contrast, Sukhi *et al *and Shashidhar *et al *argued that *D. radiodurans *exhibits the same radiation resistance even when the intracellular Mn/Fe ratio changed substantially [19,20]. Since the intracellular manganese ion level was significantly increased, a cell survival experiment was carried out to confirm whether the high intracellular manganese ion level could contribute to cellular resistance. The D_10 _value represents the irradiating dose required to reduce the population by 90%. Here, the D_10 _value was proposed to assess the resistant ability of R1 and *mntE*^- ^mutant to different stresses. As shown in Figure 5 the resistance of the *mntE*^- ^mutant under different stresses was higher than that of R1, and the D_10 _values of the *mntE*^- ^mutant were 14000 Gy γ-radiation, 700 J/m^2 ^UV, and 50 mM H_2_O_2_, whereas that for R1 was 11000 Gy γ-radiation, 600 J/m^2 ^UV, and 40 mM H_2_O_2_. Moreover, when R1 and *mntE*^- ^mutant were cultured in TGY supplemented with 50 μM manganese, their resistance to different stresses also increased remarkably, and it is consistent with their intracellular manganese level (Figure 5). The results suggest that there is a correlation between the intracellular manganese level and cellular oxidative resistance, which is consistent with the data from Daly's studies [8]. Although the role of manganese in the oxidative resistance of *D. radiodurans *remains unclear, our study implies that an increase in the intracellular manganese level may be one of the responses to oxidative stress. Moreover, it is notable that the UV resistance of the *mntE*^- ^mutant also increased. Generally, UV light results in DNA damage, and only high doses of UV cause oxidative damage. Therefore, it is interesting to speculate that the UV resistance of the *mntE*^- ^mutant may be indirectly enhanced by manganese ions. In fact, many important DNA repair enzymes use Mn^2+ ^as the cofactor [21], and manganese accumulation may have a positive effect on gene function. Furthermore, a high intracellular manganese level is also known to have an important effect on the expression of many genes including stress response genes [10].

### The mntE^- ^mutant shows a lower protein oxidation level under oxidative stress

**Figure 6:**
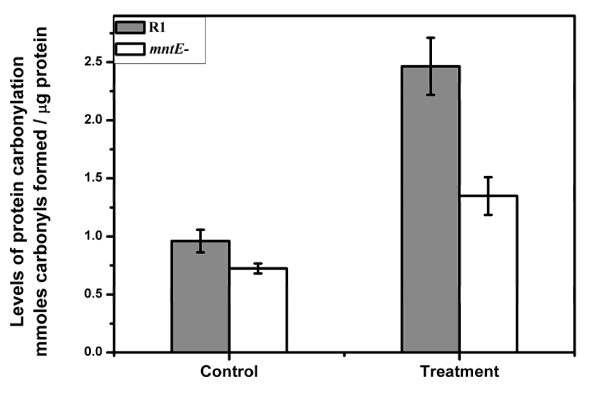
**Protein carbonylation levels in R1 (black bars) and *mntE*^- ^(white bars)**. Cells (OD_600 _= 0.8) were harvested and treated with 40 mM H_2_O_2 _for 30 min. The protein carbonylation levels were determined by the DNPH assay. Data represent the means ± standard deviations of three independent experiments.

The protein carbonylation level is an important index of intracellular oxidative damage to proteins [8]. Previous reports have shown that the proteins of IR-sensitive bacteria are more vulnerable than those of *D. radiodurans *to ROS-induced protein oxidative damage [7]. Therefore, we measured and compared the levels of protein carbonylation in the *mntE*^- ^mutant and wild-type R1. Notably, the level of protein carbonylation in the *mntE*^- ^mutant decreased to nearly 50% of that in R1 after H_2_O_2 _treatment (Figure 6), indicating that the mutation of *mntE *resulted in a lower level of protein oxidation than that observed in the wild type. This suggests that the high level of intracellular manganese ions could enhance cellular resistance by protecting proteins against ROS damage.

## Conclusions

Although it is known that the Mn/Fe ratio of *D. radiodurans *is higher than that of other bacteria, little is known regarding the maintenance of the intracellular manganese ion level in this bacterium. So far, only one manganese efflux system has been identified in bacteria [10], and it is still unknown whether this system exists in *D. radiodurans *[22]. In this study, we identified a MntE homolog in *D. radiodurans*. As expected, our results showed that the intracellular manganese ion level was almost four-fold higher in the mutant than in R1. Furthermore, we also found that the oxidative level of *mntE*^- ^proteins decreased to almost one half that of R1. On the other hand, the data also revealed that manganese accumulation is dangerous to the *mntE*^- ^mutant. Based on these data, we conclude that *dr1236 *is indeed a *mntE *homologue and is indispensable for maintaining manganese homeostasis in *D. radiodurans*. The results provide additional evidence that intracellular manganese ions are involved in the radiation resistance of *D. radiodurans*. However, because the intracellular Mn/Fe ratio and the Mn concentration of *mntE*^- ^both increased in this study, we could not clarify whether the Mn/Fe ratio or the Mn concentration is more important for stress tolerance. Therefore, global analysis of the regulation of the intracellular manganese ion level is necessary in further studies.

## Methods

### Strains and media

All the strains and plasmids used in this study are listed in the supporting information (Table 1). The *D. radiodurans *strains were cultured at 30°C in TGY (0.5% Bacto tryptone, 0.1% glucose, and 0.3% Bacto yeast extract) medium with aeration or on TGY plates supplemented with 1.2% Bacto agar.

**Table 1:** Strains and plasmids used in this study

*Strain or plasmid*	*Relevant marker*	*Reference or resource*
Strains		
*E. coli *DH5α	*hsdR17 recA1 endA1 lacZΔM15*	Invitrogen
*D. radiodurans *R1	ATCC13939	
*mntE^-^*	As R1, but *mnE*::*aadA*	This study
*mntR*	As *mntE*^- ^*mnE*::*aadA*(pME *mntE*_*Dr*_^+^)	This study
Plasmids		
pMD18-T	TA cloning vector	Takara
pRADK	*E. coli-D. radiodurans *shuttle vector carrying *D. radiodurans groEL *promoter	[27]
pME	pRADK derivative expressing *D. radiodurans mntE*	This study

### Disruption and complementation of dr1236

The mutant *dr1236 *gene was constructed as described previously [23]. Briefly, ~600-bp DNA fragments immediately upstream and downstream from *dr1236 *were amplified from the genome of the R1 strain using the primer pairs ME1/ME2 and ME3/ME4, respectively (Table 2). These two fragments were digested with *Bam*HI and *Hin*dIII, respectively, and cloned to the streptomycin-resistance DNA fragment from *pKat-aadA *[24] pretreated with the same enzymes. The ligation product was transformed into *D. radiodurans *R1, and mutant colonies were selected on TGY plates containing 8 μg/mL streptomycin. Null mutants were confirmed by PCR and sequencing, and the resulting mutant was designated *mntE^-^*.

**Table 2:** Primers used in this study

Primer Sequence	(5' → 3')
Construction of the *mntE*^- ^mutant	
ME1	GCACGCGCTTTTCCTATGAC
ME2	ATATGGATCCACCACCGCACTGAGGTATTC
ME3	ATATAAGCTTCCGGCGCCAACGTCACCATT
ME4	CGCCGACCAGGACACGATAG
Complementation of the *mntE*^- ^mutant	
ME5	ATATCATATGCCGGTTTTCGTGGCG
ME6	ATATGGATCCCAGGTCTATCAACTGTGGGA

A complementary plasmid was constructed and transformed into the *mntE*^- ^mutant as described previously [25]. Briefly, the *dr1236 *gene with the *Nde*I and *Bam*HI sites was amplified with primers ME5/ME6. The PCR product was ligated to the pMD18-T simple vector (Takara, JP), and the product was designated *pMDmntE*. After digestion with *Nde*I and *Bam*HI, the target gene MntE was ligated to *Nde*I- and *Bam*HI-predigested *pRADK *[23]. The complementation plasmid was confirmed by PCR and DNA sequence analyses and transformed into the *mntE*^- ^strain.

### Cation sensitivity assay

Cation sensitivity assays were carried out as described previously [18]. Solutions (1 M) of manganese chloride, manganese sulfate, calcium chloride, magnesium chloride, zinc chloride, cobalt (II) chloride, copper chloride, ferric chloride, and ferrous sulfate (Sigma) were prepared in milli-Q water and filter-sterilized by passing through 0.22-μm filters. Cells grown to the early stationary phase in TGY broth were plated on TGY plates and overlaid with 5-mm sterile filter discs containing 10 μL of various cation solutions. The plates were incubated for three days, and the inhibition zone of each disc was measured.

To measure the growth of *mntE*^- ^and R1, 1 × 10^5 ^cfu mL^-1 ^were grown in TGY supplemented with increasing concentrations of MnCl_2_. The OD_600 _value was measured 12 h post incubation (mean ± SD of three experiments).

### Inductively coupled plasma-mass spectrometry (ICP-MS) assay

For the ICP-MS assays [26], the cells were cultured in TGY broth that had been pretreated with Chelex (Sigma) to remove any cations and supplemented with 50 μM manganese, 10 μM ferric chloride, 100 mM magnesium, or 100 mM calcium chloride. Cells (OD_600 _= 0.6-0.8) were harvested by centrifugation, washed three times with phosphate-buffered saline (PBS) containing 10 mM EDTA, and rinsed three times with PBS without EDTA. Cells (1/10 of the total volume) were withdrawn to measure the dry weight, and the remaining cells were treated with nitric acid and used for the ICP-MS assay.

### Survival curves of the mntE^- ^mutant and R1

R1 and *mntE*^- ^cells were cultured in TGY broth with or without 50 μM manganese to OD_600 _= 1.0, centrifuged, and then resuspended in phosphate buffer. For the γ-irradiation treatment, the suspension was irradiated with different doses of ^60^Co γ-radiation for 1 h on ice. After the irradiation treatment, the cells were plated on TGY plates and incubated at 30°C for three days. The colonies were then counted. For the UV treatment, the cells were plated on TGY plates and exposed to different doses of UV radiation at 254 nm. For the H_2_O_2 _treatment, the cultures were treated with different concentrations of H_2_O_2 _for 30 min and then plated on TGY plates.

### Protein carbonylation assay

Cells grown to OD_600 _= 0.5 were treated with H_2_O_2 _(30 mM), harvested, and resuspended in PBS containing 1% (by volume) β-mercaptoethanol and 1 mM phenylmethanesulfonyl fluoride. The cells were disrupted by sonication, and the cell-free extracts were used for the protein carbonylation assay. The protein concentrations were determined by the Bradford method. The cell-free extracts were incubated with 400 μL of 10 mM 2, 4-dinitrophenyl hydrazine (DNPH) in 2 M HCl for 2 h in the dark. After precipitation with ice-chilled 10% trichloroacetic acid (TCA), the precipitated proteins were washed three times with 50% ethyl acetate in ethanol. The decolorized precipitates were evaporated and dissolved in 1 mL of 6 M guanidine hydrochloride. The solution was centrifuged, and the absorbance of the supernatant was determined at 370 nm against a protein control that had been processed in parallel but with 2 M HCl instead of DNPH. The protein carbonyl content is defined as mM/mg protein.

### Statistical analysis

Student's *t*-test was used to assess the significance between results, and *p *< 0.05 was considered as significant.

## Authors' contributions

HXS and YJH conceived and designed the study. HXS performed the experiments and wrote the manuscript. GZX, BT and HC participated in the discussion of the experimental results. HDZ and ZTS carry out the protein carbonylation analysis. All authors read and approved the final manuscript.
